# Quantitative Fluorescence Quenching on Antibody-conjugated Graphene Oxide as a Platform for Protein Sensing

**DOI:** 10.1038/srep40772

**Published:** 2017-01-13

**Authors:** Ao Huang, Weiwei Li, Shuo Shi, Tianming Yao

**Affiliations:** 1 School of Chemical Science and Engineering, Tongji University, 1239 Siping Rd, Shanghai, 200092, PR China

## Abstract

We created an immunosensing platform for the detection of proteins in a buffer solution. Our sensing platform relies on graphene oxide (GO) nanosheets conjugated with antibodies to provide quantitative binding sites for analyte proteins. When analyte proteins and standard fluorescein-labelled proteins are competing for the binding sites, the assay exhibits quantitative fluorescence quenching by GO for the fluorescein-labelled proteins as determined by the analyte protein concentration. Because of this mechanism, measured fluorescence intensity from unquenched fluorescein-labelled protein was shown to increase with an increasing analyte protein concentration. As an alternative to the conventional enzyme-linked immunosorbent assay (ELISA), our method does not require an enzyme-linked second antibody for protein recognition and the enzyme for optical signal measurement. Thus, it is beneficial with its low cost and fewer systematic errors caused by the series of antigen-antibody recognition steps in ELISA. Immune globulin G (IgG) was introduced as a model protein to test our method and our results showed that the limit of detection for IgG was 4.67 pmol mL^−1^ in the buffer solution. This sensing mechanism could be developed into a promising biosensor for the detection of proteins, which would broaden the spectrum of GO applications in both analytical biochemistry and clinical diagnosis.

Conventional enzyme-linked immunosorbent assay (ELISA) has been widely used in analytical biochemistry and clinical diagnosis. However, the accuracy of ELISA is affected by a few shortcomings intrinsic to the technique. For example, the uncertainty of the recognition between antibody and antigens could cause false signals^1^,^2^,^3^ the ELISA plates made of polystyrene lack definitive chemical surface properties to provide chemical binding of the protein^4^; and intricate procedures such as “plate cleaning” increase opportunities for systemic and human errors. In recent years, many research groups have tried to improve the accuracy of ELISA by synthesizing new ELISA polymer substitutes^4^, introducing nanomaterials^5^,^6^, employing more sensitive biomarkers^7^, and applying monoclonal antibodies^8^. All of these studies were restricted by the framework of traditional ELISA, which still relies on ELISA plates, and the problems mentioned above remain unsolved. Obviously, new immunosorbent assay techniques call for quantitative surface sites for antibody binding and fewer steps or reagents in the whole process to avoid the above-mentioned drawbacks.

Herein we present a sensing platform based on graphene oxide (GO) with definitive surface binding sites as an alternative sensing platform to that of ELISA plates. The mechanism is illustrated in Fig. 1. Human IgG was chosen as a model protein to test our design. The antibody-conjugated GO surface was able to specifically bind with both a regular IgG protein (analyte) and a fluorescein isothiocyanate (FITC)-labelled IgG protein (standard, mentioned as IgG-FITC below), thus exhibiting competitive binding of the 2 types of IgG proteins. The amount of analyte IgG protein controls the adsorption of IgG-FITC as well as the degree of its fluorescence quenching by GO. As shown in Fig. 1, when analyte IgG proteins are present to compete for the available binding sites, few IgG-FITCs adsorb on the remaining binding sites, resulting in fluorescence signals from unquenched free IgG-FITCs. Thus, the fluorescence intensity increases with an increasing amount of analyte IgG protein, given the sensing principle of our method. In the following, we briefly outline the experimental methods and then present the results in detail, followed by a discussion.

First, GO was chemically modified with rabbit anti-human IgG antibody by forming of a peptide bond between the carboxyl of GO and the amino group of the antibody. This surface provides antibody mediate binding sites that are superior to ELISA plate surfaces made of polystyrene, which rely on physical adsorption. Then, analyte IgG proteins in increasing concentrations were added to a set of buffer solutions containing antibody-conjugated GO. After adsorption of analyte IgG proteins on part of the binding sites, FITC-labelled IgG proteins with a certain concentration were also added to the above set of solutions. In this case, the GO surface not only played a role as a binding platform but also as an energy acceptor^9^,^10^,^11^,^12^ to quench the fluorescence of IgG-FITC^9^,^10^. Since quenching occurs in close proximity^13^, only the IgG-FITCs that were adsorbed on antibody-conjugated GO would be quenched^14^,^15^,^16^,^17^,^18^. As the number density of the binding sites was limited on the modified GO, more adsorbed analyte IgG proteins resulted in a stronger fluorescence signal from free IgG-FITCs in the solutions. In essence, the analyte IgG proteins control the fluorescence quenching and determine the fluorescence intensity of the whole system. Within a certain concentration range, one can generate a linear plot correlating the IgG-FITC fluorescence intensity with the analyte IgG concentration. According to our results, we achieved a limit of detection (LOD) of 4.67 pmol mL^−1^.

## Results

We first present the experimental evidence for GO surface morphology, which changes upon various stages of surface modification. We then present the quenching effect of IgG-FITC on bare GO, antibody-conjugated GO, and Bovine serum albumin (BSA)-blocked GO surfaces to characterize and optimize assay parameters. Finally, we show how analyte IgG may be detected in a wide range of concentrations based on the fluorescence signal change of IgG-FITC. In addition, we provide the limit of detection and selectivity of the assay.

Atomic force microscopy (AFM) was introduced to measure the various stages of the surface modification of GO. According to the works of Lee *et al*.^19^ and Hosseini *et al*.^4^, the height of GO would obviously increase after being activated by EDC-NHS and further upon conjugation by antibodies. These studies served as reference points for us to use AFM to characterize the surface morphologies of bare GO, EDC-NHS-activated GO, and antibody-conjugated GO to provide evidence of the EDC-NHS coupling reaction and the presence of antibodies on the GO surface.

**Figure 2:**
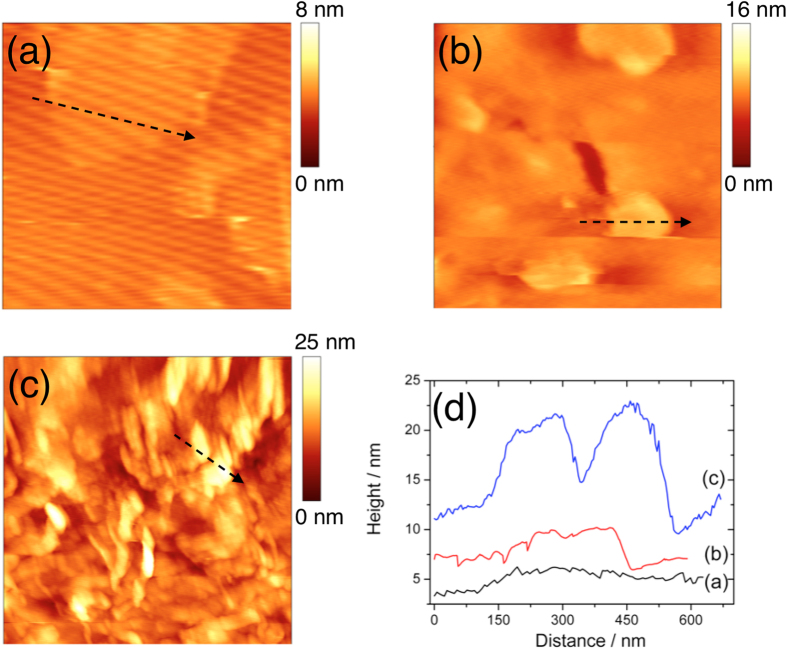
Surface morphologies of GO by AFM: (**a**) bare GO (−1 nm), (**b**) EDC-NHS activated GO (−4 nm), (**c**) antibody conjugated GO (−10 nm) on freshly cleaved mica, and (**d**) height profiles of the line scans (arrows) in (**a**), (**b**), and (**c**). Typical surface feature heights are provided in parenthesis. Image sizes: (**a**) 1 μm × 1 μm; (**b**) 1.05 μm × 1.05 μm; and (**c**) 2 μm × 2 μm.

In Fig. 2(a), bare GO has a thin and flat appearance with a height of approximately 1 nm on mica, which may correspond to the monolayer state of GO. The corresponding line scan and height profile of the sample are shown in Fig. 2(d). After activation by EDC-NHS, GO exhibits an increased thickness of about 4 nm, similar to what was reported before^4^,^19^. The line scan (arrow) gives a corresponding cross-sectional height profile in Fig. 2(d), showing a uniform surface of the GO. Because the antibody is very large (with a molecular weight of over 150 kDa), one can correctly expect that the antibody-conjugated GO should be much thicker. As shown in Fig. 2(c), we observed that the height of antibody-conjugated GO increased dramatically, with a typical thickness of about 10 nm, as suggested in the height profile in Fig. 2(d). Some bright peaks were observed on this sample, which was also similar to what Hosseini *et al*. had observed^4^. The bright peaks are likely due to the accumulation of antibodies on the GO surface. All of the surface morphology results implied that the antibody was successfully retained on the GO surface.

The key design of our immunoassay is the adsorption of IgG on antibody-conjugated GO, which controls the adsorption of IgG-FITCs and leaves some free IgG-FITCs in the solution to generate fluorescence signals. Due to the fact that binding sites on the antibody-conjugated GO were limited, when more analyte IgG proteins adsorb on antibody-conjugated GO, fewer binding sites remain for IgG-FITCs, so that more free IgG-FITCs will be in the solution and produce more fluorescence signals. Therefore, there is a positive and quantitative correlation between the analyte IgG concentration and the fluorescence intensity of free IgG-FITCs. According to this principle, our assay relies on the effectiveness of GO as an energy acceptor to provide efficient fluorescence quenching.

**Figure 3:**
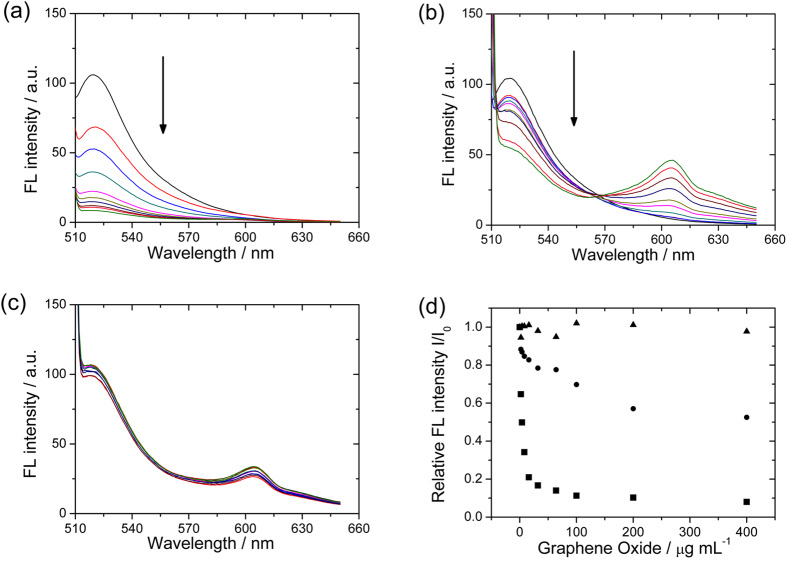
Fluorescence (FL) intensity profile of (**a**) 1 μg mL^−1^ of IgG-FITC reacting with an increasing concentration of bare GO; (**b**) 1 μg mL-1 of IgG -FITC reacting with an increasing concentration of antibody-modified GO; and (**c**) 1 μg mL^−1^ of IgG-FITC reacting with an increasing concentration of BSA-blocked GO. All of the GO concentrations varied; 0, 2, 4, 8, 16, 32, 64, 100, 200, and 400 μg mL^−1^. The relationship of the GO concentration and the FL intensity of (**a**), (**b**), and (**c**) are shown in (**d**) for comparison.

The quenching efficiency of IgG-FITC by bare GO, antibody-conjugated GO, and BSA-blocked GO was investigated by adding a variety of these 3 types of GO to standard samples containing 1 μg mL^−1^ of IgG-FITC, respectively. As we expected, the fluorescence of the free IgG-FITCs decreased when the IgG-FITCs bound to the graphene surfaces. In Fig. 3(a), a rapid decrease of fluorescence intensity was observed with the increasing amount of bare GO, exhibiting a quenching efficiency of about 90% with 10 μg mL^−1^ of GO. The signal change versus GO concentration is plotted in Fig. 3(d). The quenching efficiency of antibody-conjugated GO may be lower than bare GO, because the modification of GO with antibodies changed the electronic property of GO, changing GO to a semiconductor^19^ and making the transfer of energy from the excited state of FITC to bare GO easier than that to antibody-conjugated GO. In Fig. 3(b), the fluorescence intensity also decreased with the increasing amount of antibody-conjugated GO, and the quenching efficiency indeed dropped to about 60% at 200 μg mL^−1^, showing antibody-modified GO could still be a quencher. With 100 μg mL^−1^ of antibody-conjugated GO, about 30% of IgG-FITC initial fluorescence had been quenched, as can be seen in Fig. 3(d). Therefore, with analyte IgG in the solution, the fluorescence intensity change of IgG-FITC will be between 0 to 30% with respect to initial fluorescence with no analyte.

One last characterization of the assay was to determine whether the diminishing of fluorescence intensity could originate from other principles. If the fluorescence of IgG-FITCs will be quenched no matter if it is free or attached to the GO surface, the design is meaningless. By blocking the GO surface with 2% BSA in the buffer solution, we found that the fluorescence intensity change remained almost unchanged with increasing amount of BSA-blocked GO. Only the IgG-FITCs adsorbed on the surface of the GO could be quenched. According to our results, the appropriate concentration of antibody-conjugated GO was 100 μg mL^−1^ (~30% quenching). Although more GO might quench the fluorescence even further, a higher antibody-conjugated GO concentration could be less sensitive for sensing the analyte IgG with a very small concentration. Therefore, there is a delicate balance between the concentration of GO and the change of fluorescence signal. In the following experiment, antibody-conjugated GO always had a concentration of 100 μg mL^−1^.

Based on the above results, we carried out quantitative sensing of analyte IgG. Various amounts of IgG ranging from 0 to 20 μg mL^−1^ was prepared in the buffer solution and tested. In our experiment, the “blank” sample consisted of antibody-conjugated GO and IgG-FITC, without analyte IgG. In order to show the difference in fluorescence quenching with the addition of the analyte, we always compared the fluorescence intensity of the blank without the analyte versus that when the analyte was present.

**Figure 4:**
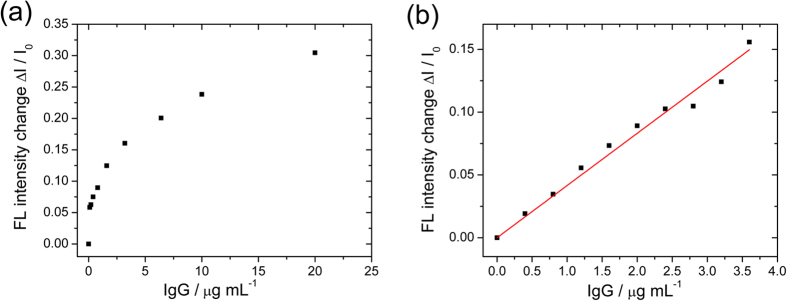
(**a**) The average fluorescence (FL) intensity change (ΔI) of 1 μg mL^−1^ of IgG-FITC as the function of the increase of the sample IgG in the concentrations of 0, 0.1, 0.2, 0.4, 0.8, 1.6, 3.2, 6.4, 10, and 20 μg mL^−1^ (experiments were performed 4 times). (**b**) The linear relationship between FL intensity change (ΔI) and low IgG concentrations of 0, 0.4, 0.8, 1.2, 1.6, 2.0, 2.4, 2.8, 3.2, and 3.6 μg mL^−1^ (experiments were performed 4 times) for the detection of the LOD. The linear fitting uses the following: ΔI = 0.04157c (concentration of IgG), R^2^ is 0.994.

As mentioned above, fluorescence quenching efficiency is determined by the energy coupling between the GO surface and the adsorbed IgG-FITC. Surface modification and interfacial environment largely affect quenching efficiency. With 100 μg mL^−1^ of conjugated GO and 1 μg mL^−1^ of IgG-FITC, our measured fluorescence intensity was ~50 at 520 nm as obtained by spectrometer. The addition of analyte IgG induced an increase of the signal, starting from the relatively large background signal. In order to make the results in direct proportion to the analyte concentration, we used the change of intensity for plotting the assay response curve in Fig. 4(a). The value of ΔI was calculated by subtracting the final fluorescence intensity at 520 nm and the “blank” sample without analyte IgG.

The experiment was repeated 4 times, and the average of the results was plotted in Fig. 4(a), from which we can see the assay’s fluorescence signal increased readily with the increasing analyte IgG concentration. There are 2 main features that can be observed from the plot. First, the signal change ΔI showed a rapid increase with analyte IgG up to ~5 μg mL^−1^, after which the change became less steep (The initial rise of the fluorescence signal was very rapid with a nearly linear region at concentrations below about 5 μg mL^−1^). Second, the signal change reached about half of the maximum value with 10 μg mL^−1^ of the analyte concentration (versus that of 20 μg mL^−1^).

According to our design, since binding sites on antibody-conjugated GO are limited, the more analyte IgG that was added, the fewer binding sites remained for IgG-FITCs, resulting in more free IgG-FITCs in the buffer solution. Thus, we observed that increasing fluorescence intensity is quantitatively controlled by the analyte IgG concentration. The result indicates that the principle of our platform is feasible, and the analyte IgG proteins could control the fluorescence quenching and determine the fluorescence intensity. Within the concentrations of 0 to 5 μg mL^−1^, we generated a linear plot relating to the IgG-FITC fluorescence intensity with the analyte IgG concentration. This is plotted in Fig. 4(b). We performed the experiment under the same conditions 4 times and the data points in Fig. 4(b) fit well with the linear function. The fitting function of the curve is ΔI = 0.04157c (concentration of IgG) with an R^2^ of 0.994.

We then repeated the blank experiment 20 times in order to calculate the standard deviation (SD). We took 3 times the value of the SD and divided it by the slope of the calibration curve, and got the LOD of 4.67 pmol mL^−1^. Up to now, we have assumed that IgG-FITC proteins are all uniformly labelled with fluorescent FITCs. Because the quality of antibodies and percentage of FITC labelling on IgG may vary in different batches and from different manufacturers^20^, the LOD could in principle be improved with well-controlled and uniform labelling of IgG by FITC. However, this is beyond the scope of this paper.

**Figure 5:**
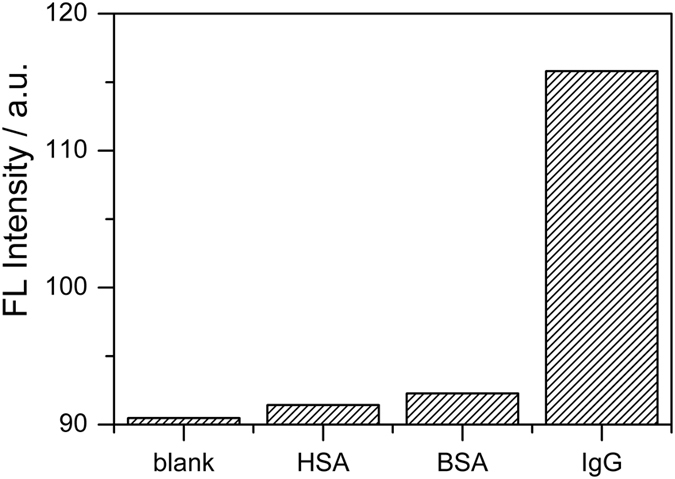
Selectivity. The concentrations of HSA and BSA were both 10 μg mL^−1^, the IgG-FITC was 1 μg mL^−1^, and the antibody-conjugated GO was 100 μg mL^−1^ for each experiment. The blank sample did not contain analyte IgG or an interfering molecule.

One major assumption we held is that IgG proteins bind on antibody-conjugated GO via the antibody-antigen type of specific bonding. This discriminative binding process means that analyte IgG will be adsorbed by antibody-conjugated GO, preventing the fluorescein-labelled IgG-FITC from binding to and being quenching by the surface. In order to know whether other biological molecules might interfere with the sensing process and give false positive signals, we performed a series of experiments to determine more about the specificity of our assay. Some interference molecules, such as human serum albumin (HSA), BSA, and IgG, were introduced to test the selectivity. In this set of parallel testing, the signals from the solutions containing the above interference substances, analyte IgG, and blank sample without analyte IgG were compared. The results in Fig. 5 clearly show that the 3 possible interfering biomolecules gave no detectable signal change, as compared with the blank sample. Only the analyte IgG protein showed a significant positive signal as the fluorescence intensity of the other proteins was close to the blank sample. This indicates that our assay design is highly selective for IgG protein.

## Discussion

We established an immunosensor platform based on quantitative fluorescence quenching between fluorescein-labelled antigens and antibody-conjugated GO nanosheets, a process controlled quantitatively by the concentration of analyte proteins. Human IgG, a widely used model protein, was chosen to test the feasibility of our design. With the competitive binding of analyte IgG and standard IgG-FITC the surface of GO’s limited binding sites, the process conveniently paves a way to control free IgG-FITC in the solution by analyte IgG adsorption on the binding sites. The increase of fluorescence signal from IgG-FITC correlates directly with an increasing analyte IgG concentration. The assay based on antibody-conjugated GO does not require any phase separation steps or wash steps as with commercial ELISA procedures, which are used to remove unadsorbed antibodies. Thus, our assay is beneficial since it uses fewer reagents, has a lower cost, and there are fewer opportunities for systematic and human errors. The LOD was 4.67 pmol mL^−1^, which still leaves room for further improvement. The sensing mechanism in this study could become a viable immunosensor platform for the detection of proteins, which will broaden the spectrum of graphene oxide applications in both analytical biochemistry and clinical diagnosis.

## Methods

### Materials

BSA was purchased from Solarbio (Beijing); IgG, IgG-FITC, and rabbit anti-human IgG antibody were obtained from Sangon Biotech Co. Ltd. (Shanghai); graphene oxide was bought from XFNANO (Nanjing); and N-(3-dimethylaminopropyl)-N-ethylcarbodiimde hydrochloride (EDC) and N-hydroxysuccinimide (NHS) were purchased from Tokyo Chemical Industry Co. Ltd. (Japan). They were all of analytical reagent grade and used without further purification.

### Characterization Methods

The fluorescence intensity of each sample was measured under the excitation wavelength of 490 nm, with the slit width of 10 nm, and voltage of 500 V using a F-7000 fluorescence spectrophotometer (Hitachi, Japan). The emission peak of FITC was centered at 520 nm, and corresponding filters were introduced to obtain the analyte spectral region. For AFM measurements, sample solutions containing bare GO or modified GO were dripped on freshly cleaved mica using a pipette and then dried in ambient air. Surface topographic features were scanned in contact mode using a commercial AFM (CSPM 4000, Benyuan, China) equipped with a silicon cantilever.

### Preparation of Rabbit Anti-human IgG Antibody-conjugated Graphene Oxide

Antibody-conjugated GO was synthesized by a classic two-step EDC-NHS (1-Ethyl-3 -(3- dimethylaminopropyl) carbodiimide and N-hydroxysuccinimide) method^21^,^22^,^23^. Briefly, 1 mg of GO was ultrasonically dispersed in 5 mmol of L^−1^ 2-(N-Morpholino) ethanesulfonic acid (MES) buffer (pH = 4.0). Then a MES buffer solution containing 4 mg mL^−1^ of EDC and 6 mg mL^−1^ of NHS was added into the GO-dispersed MES solution to activate the GO. The mixture was first stirred for 30 min at 15 °C, then centrifuged and washed with 20 mmol L^−1^ of phosphate buffer solution (PBS, pH = 7.4) 3 times to remove unreacted coupling reagents. The activated GO was redispersed in PBS to react with 50 μg of rabbit anti-human IgG so as to modify the GO with the antibody. The samples were mixed on an electronic shaker at 15 °C for 2 h. Remaining active sites of GO were blocked with 2% BSA in 20 mmol L^−1^ of PBS buffer solution for 30 min. The solution was then centrifuged at 16,000 rcf for 10 min to remove any unbound biomolecules in the supernatant.

### Process for IgG Protein Assaying

For a typical procedure of analyte IgG sensing, a series of concentrations of human IgG as the analyte ranging from 0–20 μg were separately added into these tubes and reacted for 1 h at 37 °C. Afterward, human IgG-FITC standard was added to each sample to reach a concentration of 1 μg mL^−1^ and allowed to react for another hour at the same temperature. When the reaction ended, the fluorescence intensity of each sample was measured. The response curve of the assay was obtained by plotting the IgG-FITC’s fluorescence intensity change as a function of the analyte IgG concentration. Methods for determining the LOD and selectivity of the assay were essentially the same as the above processes.

## Additional Information

**How to cite this article:** Huang, A. *et al*. Quantitative Fluorescence Quenching on Antibody-conjugated Graphene Oxide as a Platform for Protein Sensing. *Sci. Rep.* **7**, 40772; doi: 10.1038/srep40772 (2017).

**Publisher's note:** Springer Nature remains neutral with regard to jurisdictional claims in published maps and institutional affiliations.

## Figures and Tables

**Figure 1:**
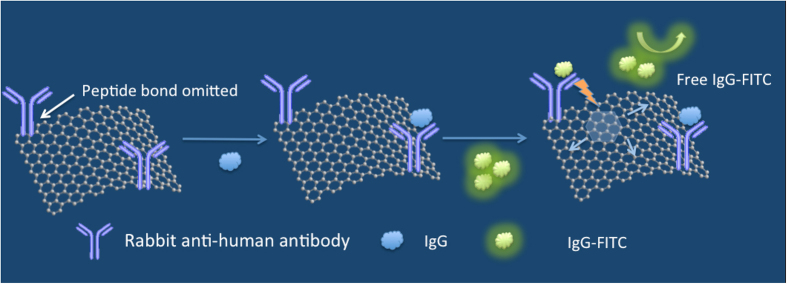
Graphene oxide-based fluorescence quenching for the detection of IgG proteins. The more analyte IgGs were adsorbed by the antibody-conjugated graphene oxide, the fewer IgG-FITCs were quenched. The fluorescence signal from free IgG-FITCs in the buffer solution correlates with the analyte IgG concentration.
